# Inclusion of person-centred care in UK postgraduate medical education curricula: Interviews and documentary analysis

**DOI:** 10.1186/s12909-023-04730-2

**Published:** 2023-10-11

**Authors:** Heather L. Moore, Rose Watson, Allison Farnworth, Karen Giles, David Tomson, Richard G. Thomson

**Affiliations:** 1 School of Psychology, Newcastle University, Newcastle Upon Tyne, NE2 4DR UK; 2 School of Education, Communication and Language Sciences, Newcastle University, Newcastle Upon Tyne, UK; 3 Population Health Sciences Institute, Newcastle University, Newcastle Upon Tyne, UK; 4 Faculty of Health Sciences & Wellbeing, University of Sunderland, Sunderland, UK; 5 Collingwood Surgery, Collingwood Health Group, North Shields, UK

**Keywords:** Person-centred care, Postgraduate education, Curriculum, Medical

## Abstract

**Background:**

Person-centred care (PCC) involves placing people at the centre of their healthcare decision making to ensure it meets their needs, values, and personal circumstances. Increasingly, PCC is promoted in healthcare policy and guidance, but little is known about how this is embedded in postgraduate medical training. The aim of this research was to understand how PCC is embedded in UK postgraduate medical training and explore factors influencing inclusion of PCC in curricula content.

**Methods:**

To explore this, we interviewed senior professionals with key roles in the curricula from four UK Royal Colleges (Psychiatrists; Physicians; Surgeons; and GPs) and used framework analysis on interviews and relevant curricula documents to identify themes.

**Results:**

Legislation and professional/educational guidance influenced inclusion. PCC definitions and terminology differed and placement within curricula was variable. Royal Colleges defined the curriculum and provided training to ensure competence, but local deaneries independently implemented the curriculum. Trainer engagement was greater than trainee buy in. Quality assurance focused on feedback from trainers and trainees rather than patients, and patient and public involvement in curriculum development, teaching, and assessment was limited.

**Conclusions:**

There is a need for cross-organisation collaboration to develop a PCC competence framework that defines the skills and level of competence required at different points in training, with clarity around the differences between undergraduate and postgraduate requirements. Greater auditing and quality assurance of programme delivery would help identify successful practices to share within and across Royal Colleges, while still maintaining the flexibility of local provision. Engagement with patients and the public in this work can only strengthen provision.

**Supplementary Information:**

The online version contains supplementary material available at 10.1186/s12909-023-04730-2.

## Introduction

Person-centred care (PCC) is a well-recognised concept in contemporary health care that involves putting people at the centre of their healthcare decision making to ensure it meets their needs, values, and personal circumstances [1–5]. PCC incorporates strategies such as shared decision making (SDM), supported self-management, and personalised care planning. It has been demonstrated internationally, and across a range of interventions and measured outcomes, that PCC has multiple positive impacts. Taking a PCC approach to healthcare delivery has been associated with improved health outcomes, better knowledge and understanding of risk, more active involvement in decision making and decisions better aligned with patient values, greater adherence to recommended clinical practice and medication, and reduced rates of elective procedures [6–9]. Furthermore, SDM training for registered professionals has been shown to have impacts on both patient and practitioners. For example, it has been linked to improved patient satisfaction, reduced patient anxiety and improved treatment adherence [10, 11]. For practitioners, there have been improvements in professional decision coaching skills (including assessment of decisional needs, such as information, values clarity, support, stage and timing of decision) [12], both observer- and self-assessed communication [13], and perceived involvement of patients in decision making [14].

Despite this, evidence suggests implementation of PCC practices in health care settings is variable and challenging [15, 16]. There is, for example, a paucity of evidence addressing the effectiveness of interventions aimed at improving healthcare professionals’ uptake of SDM. Interventions targeting both healthcare professionals and patients appear most promising [16–19] but variability in outcomes evaluating the impact of training programmes creates a challenge for comparing the relative effectiveness of programmes across studies [20, 21].

Increasingly, PCC education and training has been promoted by bodies involved in healthcare policy and guidance on delivery (e.g. [4, 22–25]). A major SDM implementation programme (Making Good Decisions in Collaboration; MAGIC) highlighted the importance of embedding PCC, and SDM micro-skills training, in educational curricula to improve delivery of PCC in healthcare professionals’ everyday practice [15]. However, very little is currently known about the extent to which PCC is embedded in UK postgraduate medical training, a fact that is complicated by the independence of Royal Colleges in development of their required training provision. This work focuses on UK postgraduate medical education, as part of a wider training needs analysis (TNA) that also included undergraduate training (reported separately [26]). We aimed to understand how PCC is represented in postgraduate curricula across UK Royal Colleges, and to explore factors influencing inclusion of PCC in curricula content.

## Methods

Inclusion criteria were medical Royal Colleges in England. Each College was approached by email to participate in the TNA and all Royal Colleges that were approached agreed to participate. We chose four medical Royal Colleges in England as exemplars in this TNA: Psychiatrists (RCPsych); Physicians (RCP); Surgeons (RCS); and General Practice (RCGP). They were chosen to give a range of specialities covering both primary and secondary care, as well as incorporating subspecialty elements, allowing representation across a range of Royal Colleges. As a preliminary step, a rapid literature review was conducted to gather definitions of PCC and identify framework coding themes [1–3, 23–25, 27–38]. PCC elements generated from this exercise included: planning; coordination; shared decisions; health literacy; self-management; communication; and involvement.

Two methods were then used to explore postgraduate curricula content: (1) telephone interviews with key informants, and (2) documentary analysis of curricular documents.

### Interview data

Each College identified the most appropriate informant to participate in this research, resulting in four informants (one per College) overall. They were all senior professionals with a lead role for curricula in each College (e.g. medical directors, associate deans, programme leads). PCC elements identified in the rapid literature review guided development of open-ended questions, and pre-specified prompts for the schedule [39]. Questions centred around inclusion of PCC, influences on the curriculum, barriers and facilitators to inclusion, quality assurance, and patient and public involvement (see Appendix for schedule). The researcher and interviewee had contact prior to interview to arrange the interview but did not otherwise know each other. All interviews were conducted over telephone with only the researcher (AF or KG) and interviewee present, and audio recorded with the informed consent of the interviewee (except one failed recording where the researcher took contemporaneous notes). Interviews lasted around 1 h. Both interviewers were female, came from a nursing background (AF with an additional PhD in health service quality, KG with an additional MSc in health services management), and were employed as researchers at the time of the study. Interviews were transcribed verbatim and the transcripts, plus interviewer notes, formed the data for analysis.

### Curriculum documents

All Royal Colleges except RCGP operate with a ‘core’ curriculum and ‘specialist’ curricula; the number of specialisms operating within each College varied (Table 1).

**Table 1:** Core and specialist curricula time allocation

	Core/Run Through Training	Speciality Training	Number of Specialities
Royal College of Psychiatrists	3 years	3 years	8
Royal College of Physicians	2/3^a^ years	5/4^a^ years	33
Royal College of Surgeons	2 years	4 years	10
Royal College of General Practitioners		3 years	

^a^Interviewee indicated that the structure of the training was due to change to incorporate an increase in the time allocated to ‘core’ training and a concomitant decrease in the time spent on speciality training

Curriculum documents were obtained from Royal College websites; the core curriculum for each Royal College, and additionally, one speciality training curriculum document for each of RCS and RCP were reviewed (Table 2), in order to follow through the analysis in these two Colleges that have many subspecialties. The documentary analysis used a framework of PCC elements identified by the rapid literature review.

**Table 2:** Curricula documents reviewed

Royal College	Core Curriculum	Speciality Curriculum
RCPsych	Royal College Psychiatrists (2013). A Competency Based Curriculum for Specialist Core Training in Psychiatry. Core Training in Psychiatry CT1-CT3 [40]	
RCP	Joint Royal College of Physicians Training Board (2009). Speciality Training Curriculum for Core Medical Training [41]	Joint Royal College of Physicians Training Board (2010). Speciality Training Curriculum for Cardiology [42]
	Intercollegiate Committee for ACCS Training (2012). Acute Care Common Stem Core Training Programme. Curriculum and Assessment system [43]	
RCS	Intercollegiate Surgical Curriculum Programme (2015). Core Surgical Training [44]	Intercollegiate Surgical Curriculum Programme (2014). Cardiothoracic Surgery Curriculum (and congenital cardiac surgery sub-speciality) [45]
RCGP	Royal College of General Practitioners (2016). The RCGP Curriculum: Core Curriculum Statement. 1.00: Being a General Practitioner [46]	
	Royal College of General Practitioners (2016). The RCGP Curriculum: Professional and Clinical Modules in 2.01–3.21 Curriculum Modules [47]	

### Analysis

We employed a deductive approach in order to map the curriculum and practice on to PCC guidance. Nvivo version 12 [48] was used to manage the interview data. All available interview data were analysed. The data were analysed using the principles of framework analysis [49] to organise the data into pre-defined categories relating to PCC inclusion in the curriculum. Different frameworks were designed from the rapid review and applied to the interview and curricula analyses (see Supplementary Table 1 and 2), to understand 1) How the guidance is applied across curricula, and 2) How curricula are enacted in practice. During interview analysis, an additional code was identified and incorporated into the analytical framework (Supplementary Table 1).

Two researchers independently analysed the data and met regularly to ensure consistency in coding and to resolve any variation in the interpretation of the data. AF’s background is described above, and RW is a medical sociologist with an MSc in social research. The interviews and documentary analyses were conducted independently, and following individual analysis, findings were compared to identify common emergent themes. Inter-related ideas and emergent themes within the data were identified.

## Results

**Fig. 1:**
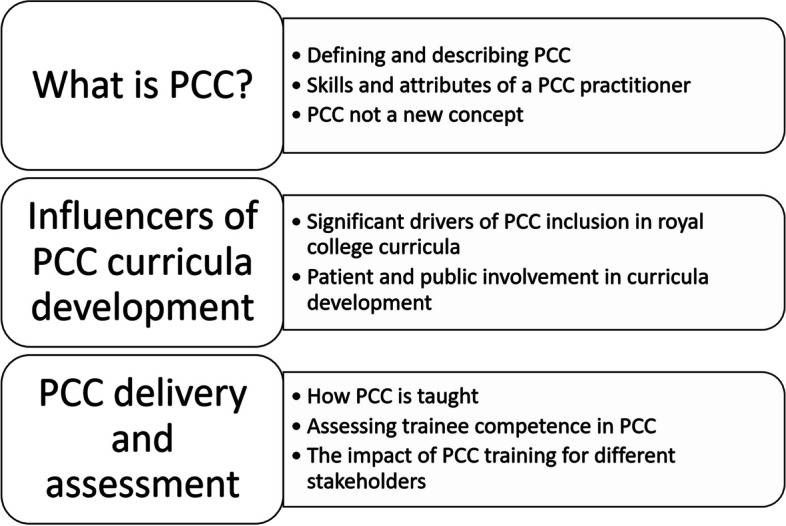
Superordinate and subordinate themes resulting from integrative analysis of Royal College interviews and curricula documents

Three superordinate themes emerged during integrated analysis of the interviews and curricula documents: (1) What is PCC?; (2) Influencers of PCC curricula development; and (3) PCC delivery and assessment. Each superordinate theme split further into subordinate themes (Fig. 1), which we discuss in turn below.

### Theme 1: What is PCC?

#### Defining and describing PCC

Reference to PCC during interviews and within curricula documents was often general, with little definition or description of what PCC might mean in practice (Table 3a and b), although the RCGP curriculum offered a more comprehensive definition (Table 3c). However, there was a feeling among interviewees that PCC can be a broader concept, incorporating, for example, recovery-based models of healthcare, reflective practice, therapeutic relationship building, holism, personalised care, and enablement (Table 3d).

**Table 3:** PCC definitions obtained from interviews and curricula documents

a) “patients are the central focus of care comes up a lot” (RCP)
b) “Work with stakeholders to create and sustain a patient-centred service”, Cardiothoracic surgery curriculum, Intercollegiate Surgical Curriculum Programme (ISCP), 2014 [45]
c) RCGP definition of PCC:
1. Understanding the wider context of the consultation: this means perceiving that your patient is a person; a belief that the sick patient is not a broken machine; and that ‘health’ and ‘illness’ comprise more than the presence or absence of signs and symptoms. A constant willingness, therefore, to enter your patient’s ‘life-world’ and to see issues of health and illness from a patient’s perspective, considering social, educational and cultural differences
2. Recognising that patient-centred medicine depends on an understanding of the structure of the consultation – in particular that good consultations are often associated with particular consultation styles and skills. However, the expectations and preferences of your patients will vary, so that as a patient-centred doctor you must be able to select from a range of styles and skills
3. Being committed to an ethical, reflective attitude that enables you to understand and monitor your practice, and develop it to the benefit of your patients *The RCGP Curriculum: Professional and Clinical Modules, p5-6″ *[47]
d) “reflective practice, there are a few different ways of viewing that, you could just think about it as a self-improvement tool but reflective practice also means being able to reflect in the, in the moment with the patient, you know” (RCPsych)

It is also noteworthy that “patient-centred” rather than “person-centred” care is the term most widely used in the documents reviewed.

#### Skills and attributes of a person-centred practitioner

Most interviewees highlighted the importance of communication skills for delivery of many aspects of PCC (Table 4a). The curricular analysis also found a broad range of statements coded under communication. *Communication, partnership and teamwork* is one of the four domains of Good Medical Practice [50], a General Medical Council (GMC) document designed to support the curriculum, which was referenced in each of the curricula documents. Here, a clearer definition is provided, linking communication and person-centredness (Table 4b).

**Table 4:** Proposed skills and attributes of a person-centred practitioner

a) “Well the professional skills sort of fits the specialty knowledge and skills syllabus and it covers things like communication with patients, communication with colleagues as well, but communication with patients so being able to strike up a rapport, listen actively, respond compassionately, check the patient’s understanding, involve the patient in their healthcare, taking opportunities to help patients care for themselves, that sort of thing < … > the professional skills syllabus is about being a good communicator and within that sort of working with patients, I think that is the main area” (RCS)
b) Communication, Partnership and Teamwork Domain of Good Medical Practice (50; p13):
1. You must listen to patients, take account of their views, and respond honestly to their questions
2. You must give patients the information they want or need to know in a way they can understand. You should make sure that arrangements are made, wherever possible, to meet patients’ language and communication needs
3. You must be considerate to those close to the patient and be sensitive and responsive in giving them information and support
4. When you are on duty you must be readily accessible to patients and colleagues seeking information, advice or support
c) “psychiatry is, by its very nature, a speciality where empathy and a person-centred message is part of everything we do” (RCPysch)
d) “As a GP, this means you should < … > Demonstrate a non-judgmental approach in your dealings with patients, carers, colleagues and others, respecting the rights and personal dignity of others and valuing diversity” (46; p15)
e) “no, it doesn’t use those words [shared decision making] and maybe there is room to actually use that particular phrase, < *…* > but it might say a behaviour is to ‘demonstrate an inclusive and patient-centred approach, with respect for the diversity and values in patients, carers, and colleagues’. That is one of the behaviours that is expected within this topic, so that is probably as far as it goes” (RCS)

Interviewees described other associated skills and attributes that were key to a person-centred practitioner, including empathy, compassion, and a non-judgemental attitude (Table 4c and d). Specific skills associated with PCC identified by interviewees included SDM or personalised care planning; however, there was variation in the wording used to describe these skills (Table 4e).

‘Coordination’ was a theme well covered in curriculum documents and linked strongly to ‘communication’. The RCGP, under their section *‘Enable people living with long-term conditions to improve their health’,* particularly emphasises *“the harm to a patient's health and the costs to the health service that arise when care is inappropriate, fragmented or uncoordinated” *[46].

#### PCC is not a new concept

Interviewees felt that PCC is not a new concept and that their specialisms were already underpinned by philosophies aligned to PCC; indeed, the RCGP curriculum uses language which implies that PCC forms an integral part of what GPs do [46]. However, specialities do not all describe PCC using the same terminology, meaning it may not be recognised by those outside of the speciality (Table 3). Further, the RCPsych interviewee identified challenges to teaching PCC without implying that their clinicians lacked these skills.

“I think sometimes people get a bit defensive because they think ‘well, I’ve been doing this, and then you’re telling me I’m not practising in a person-centred way’ <…> and where people are doing it very well but not calling it person-centred, that’s fine, you know, we don’t want to denigrate them in any way” (RCPsych)

### Theme 2: Influencers of PCC curricula development

#### Significant drivers of PCC inclusion in Royal College curricula

**Fig. 2:**
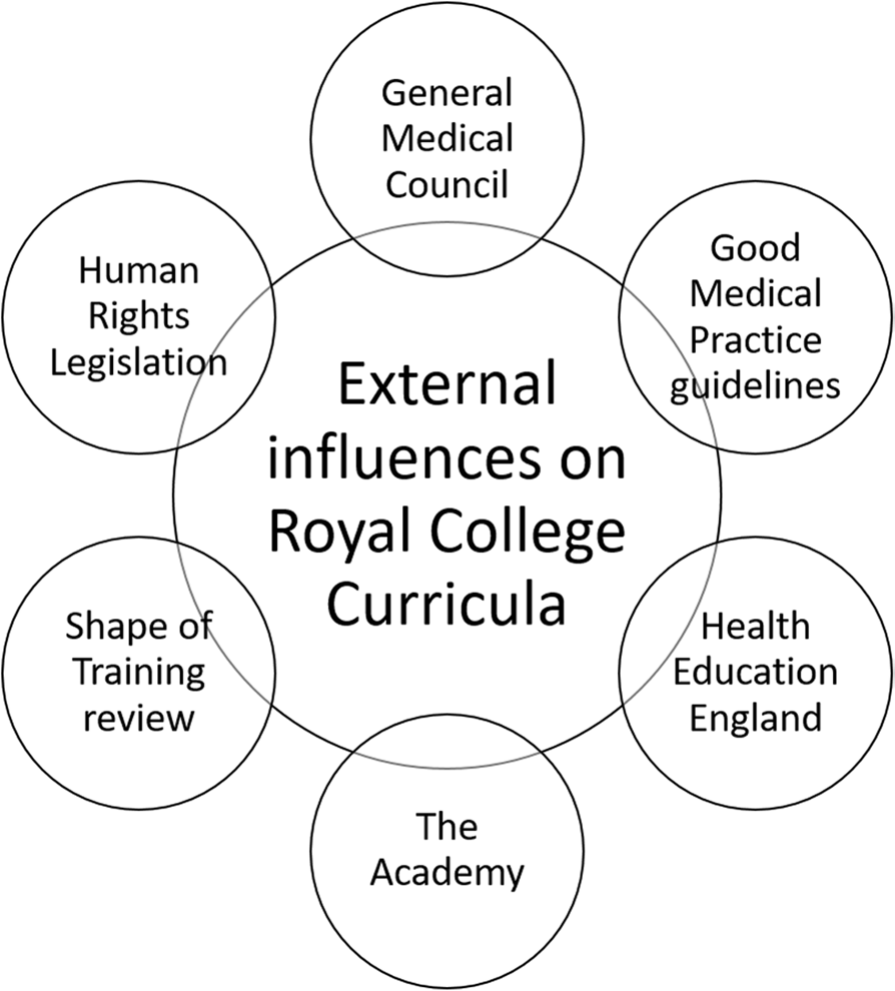
External influences of PCC inclusion within the Royal College curricula

Figure 2 shows external drivers of PCC inclusion. Naturally, the GMC and their guidelines were key, and inclusion of PCC can be clearly seen in these guidelines (Table 4b). Other examples provided did not always explicitly reference PCC (e.g. Human Rights legislation). It was noted by one interviewee that policy amendments can demand changes which are difficult to fit within an already crowded curriculum (Table 5a).

**Table 5:** Internal drivers of PCC inclusion

a) “In response to the question about national priorities and policies, and the impact on their programme, it became evident that this is a frequent issue, impacting on their annual review processes. He made reference to the challenge of fitting in all the relevant demands into the three-year programme.” (Fieldnote, RCGP interview)
b) “ < … > values based practice < … > compassionate practice, or recovery focused practice which I think all occupied the same perceptual space, which is probably the same perceptual space that you’re talking about, which is about, shared decision making and collaborative practice, person-centred practice. So, I set up a group, at college last year, < … > and the group essentially looked at person-centred care in the curriculum, because for me I think the issue was I do believe that education underpins the care we provide, and so we need to make sure that person-centred training is right” (RCPsych)
c) “we have done a very large piece of work on shared decision-making, supported self-management out of that. It was one of the strong recommendations in the Future Hospital Commission Report. < … > So, that’s part of what has become the Future Hospital programme, and the Future Hospital programme has had a number of manifestations” (RCP)
d) “the challenges are always to do with communicating and getting it across to people, < … > but generally, if these things are adopted across all the specialities, and they are discussed with all the specialities, then that is quite easy to get across and get approved [by the GMC] as well” (RCS)

Alongside external influences, interviewees described PCC promotion within their respective Royal Colleges, driven by methods such as simulation-based human skills training and PCC special interest groups (Table 5b). The RCP completed a significant consultation resulting in a report about the provision of acute care (the Future Hospital Commission Report [51]) which emphasises the importance of SDM and integrated care and has driven their practice (Table 5c). A commonly identified internal barrier to curricula change was the difficulty achieving consensus when working with a number of specialities that must all agree before action can be taken. However, this could also be a strength in terms of implementation, where proposed changes were supported (Table 5d).

#### Patient and public involvement in curricula development

Despite the person being the main focus of PCC, there was little evidence of patient or public involvement in curricula development, training or assessment of PCC skills in either interviews or curricula documents. Consistent with this, the Royal College curricula websites offer very limited guidance about how patients and public can be involved in developing PCC curricula or assessing competence. Interviewees noted that patients and/or the public were usually represented on their committees. There was, however, little to show how influential they are to the development and/or assessment of the curricula and no specific examples of patient and/or public involvement as substantial drivers in curricula development were given (Table 6a and b). Nevertheless, some interviewees identified direct patient involvement in trainee assessment using surveys or including patients in multi-source feedback methods (Table 6c and d).

**Table 6:** How are patients and the public involved in Royal College curricula development?

a) “whenever a curriculum change is made we always involve representatives of patients and their groups, but we also have a wide range of stakeholders who we take feedback from” (RCS)
b) “In terms of getting patient feedback on how it works, I don’t think we’ve gone down that route yet, and I’m not sure just how there has been patient involvement in the curriculum, because we’ve had patient representatives on all our committees at the College, we have quite a large, and very active, patient and carer network at the College and they are intimately involved” (RCP)
c) “Yes, so all trainees, even at the moment, have to [formal patient feedback] questionnaire process [at least] once or twice during their higher medical training.” (RCP)
d) “He noted that the trainees also do an MSF, Multi-Source Feedback, and when they are in a GP practice placement, have patient feedback through a patient satisfaction survey.” (Fieldnote, RCGP interview)

### Theme 3: PCC delivery and assessment

#### How PCC is taught

All interviewees suggested PCC is a key part of contemporary healthcare and is therefore included in the curricula of healthcare professional training programmes. Generally, interviewees felt that PCC underpinned their specialisms but that it may not be obvious due to terminology.

**Fig. 3:**
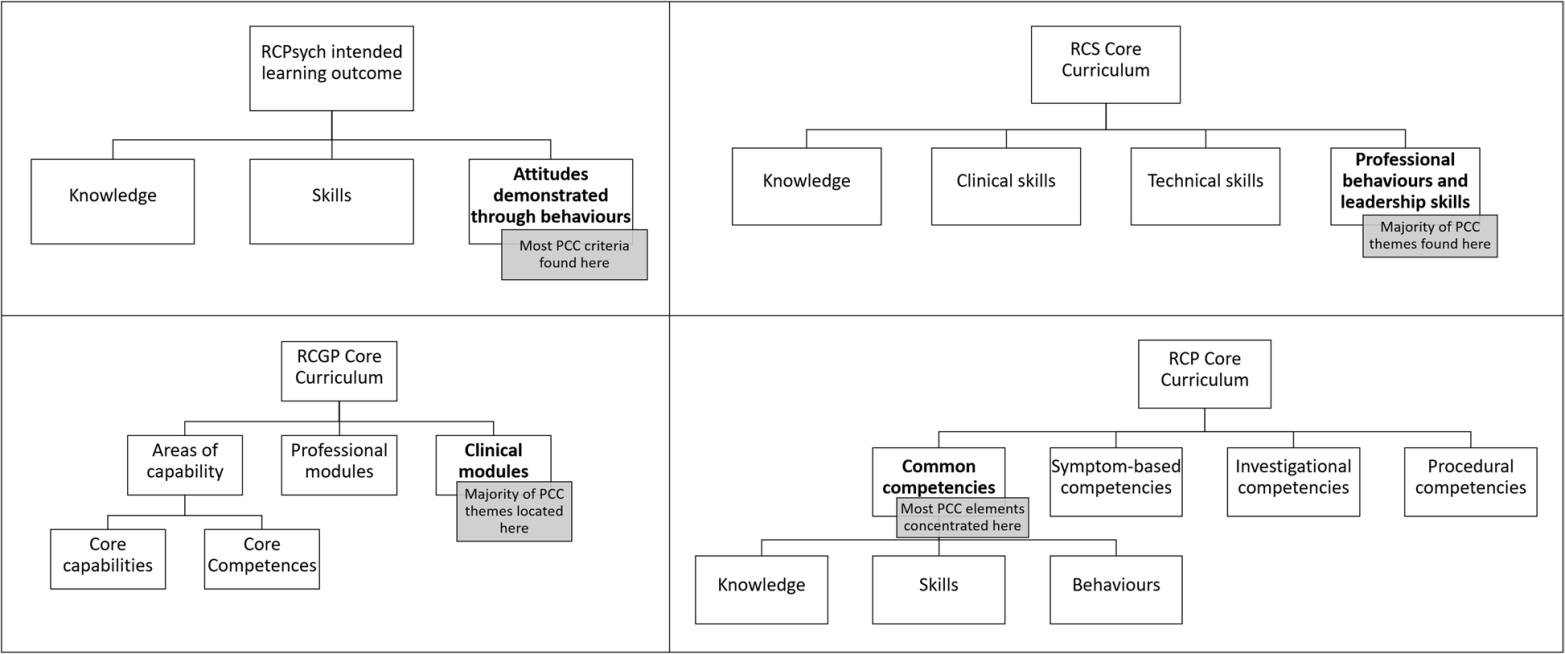
Structure of curricula and location of majority of PCC elements within each Royal College. N.B. These flow diagrams represent the basic rather than detailed structure. Level 2 of the hierarchy shows the divisions in training area for each Royal College; the bold headings highlight where the majority of PCC-specific elements were found. For RCP, the level 3 subdivisions are illustrative of the subdivisions of each strand of the level 2 divisions in training

The location of PCC-specific content within curricula varied between Royal Colleges (Fig. 3). RCPsych and RCS placed the majority of their PCC teaching within sections relating to professional skills, whereas RCP and RCGP housed their PCC content within sections covering specific clinical areas such as long-term conditions and end of life care. Although most RCPsych PCC content featured under professional skills, they also integrated PCC specifically into teaching about long-term conditions.

Interviewees indicated that PCC training was integrated across all years, with increasing complexity in teaching and competence of trainee knowledge, attitudes, and decision making expected at each stage, described as a ‘spiral’ curriculum by RCPsych and RCP [40, 52]. How this approach applies to PCC training is evident within specific sections in RCP General Internal Medicine curriculum, where the level descriptors for ‘managing long term conditions and promoting patient self-care’ show increasing levels of competency relating to PCC, from “Is aware of the need for promotion of patient self care” at level 1 to “Helps the patient networks develop and strengthen” at level 4 [52]).

Whilst the Royal Colleges play a strong role in defining curriculum and trainee competence requirements, local deaneries implement training independently (Table 7a), potentially leading to variation in practice (Table 7b). Interviewees indicated that they do not directly assess the specific content of PCC teaching by local deaneries; however, they do implement training for trainers in their deaneries to ensure trainer competence (Table 7c).

**Table 7:** Practical Implementation of PCC Curricula Training

a) “the local deanery decide how they roll it out, we have a structure where trainees have to have a programme director and a named signed educational supervisor and clinical supervisor who will be involved in training, but how and when these things actually happen are all determined locally. We also leave it to the various education departments, whether it is within a deanery or whether it is within colleges and faculties, to teach, you know to run courses and that sort of thing.” (RCS)
b)“each Deanery, and in the denominations, their local versions of the Deaneries, they provide courses, for example, and the curriculum is covered in the course, I think it’s probably fair to say that there is a fair amount of regional variation in how the teaching happens, they all are teaching to the same syllabus and the curriculum, but how it’s actually done, in practice, does vary.” (RCPSych)
c) “But we expect that anyone who is a named supervisor will be suitably qualified, will have attended certain training courses and will have an appraisal with a line manager to look at how they are performing and then through the ARCP [Annual Review of Competence Progression] we also find out whether there is evidence that it is working and where the problem areas are” (RCS)

#### Assessing trainee competence in PCC

Regarding assessment, interviewees described techniques that involve practical demonstration of skills as the most relevant way to assess PCC competence (e.g. by using workplace assessments (Table 8a)), although no specific examples of this were found within the curricular analysis. PCC was indirectly assessed in written exams (Table 8b). There was variation in the extent to which interviewees felt that their assessment processes adequately measured trainee PCC competence, with the RCPsych interviewee reflecting on uncertainty about how soft skills, including PCC, are assessed (Table 8c).

**Table 8:** Strategies and barriers for assessing PCC competence

a) “there is an assessment system, then exams and workplace based assessments which all follow the curriculum and trainees are expected, when they do workplace assessments to link specifically to various syllabus topics and then in the exam there will be exam questions on various different areas of all the syllabuses.” (RCS)
b) “in the final exam, < … > I don’t think < … > that there is a specific, set of questions that cover person centred care, but, having said that, I am sure that there are questions that probably map onto that territory, < … > questions around historical aspects of psychiatry and the potential for psychiatry to have been abused for example, so, so there might be something along those lines that touch upon the topic” (RCPsych)
c) “It has been difficult to think about how we actually assess person-centredness, < … > I guess it is a generic issue about how do we assess things like empathy, relationship building, you know, values-based practice, so it’s a challenge for summative exams to do that in the short time and, sort of, artificial environment exams often are in” (RCPsych)
d) “the curricula are underpinned by competences, and there are millions of them, and assessing those competences is very time-consuming, quite onerous, and actually, to be honest with you, a lot of the stuff that’s done around assessing individual competence is not necessary. And it’s burdensome, and actually, sometimes, not terribly meaningful. Plus, the competences are very trainee-focused. You know, “Can the trainee pinch the left big toe?” < … > What you really want to know is how the trainee can look after a patient” (RCP)
e) "It basically tests competences by asking a slightly different question. What would I trust this trainee to do, and how could I break down their practice in a meaningful way, where I can ask that question? So, we have used the term ‘CIPs’, C-I-P-s, Competences in Practice” (RCP)
f) “He gave some examples, and said that they were certainly seeing some paternalism in some trainees, which potentially could be attributed to their culture and their race. This resulted in them perceiving themselves as the expert, as opposed to the patient. They had seen this in watching trainees through the OSCE [Objective Structured Clinical Examination] process, and this was noted in a number of overseas trainees. They have done a significant amount of work to try and identify differential attainment, to assess it, give appropriate feedback, and then address it.” (Fieldnote, RCGP interview)

Another interviewee described that competencies have been historically focused on trainee competence in a number of discrete skills, without necessarily considering how the trainee manages those skills together in practice (i.e. to deliver care in a PCC way; Table 8d). To tackle this issue, the RCP interviewee described how they had repositioned their assessment criteria to focus more holistically on the specific skills required to practice in a person-centred way (Table 8e). Developing trainees who are not perceived to meet competency requirements represented a further challenge, with descriptions about identifying specific areas trainees can improve upon. One interviewee explained that their College had identified cultural factors that impact on trainee attitudes, behaviours and communication skills. Implementing a strategy to develop trainees who do not meet PCC competency had therefore become a priority for this College (Table 8f).

#### Quality Assurance of PCC in the Curriculum

Royal Colleges do not have direct oversight of the ways that local deaneries meet trainee competence requirements for PCC in their teaching (Table 9a). However, all interviewees described quality assurance feedback mechanisms, such as: quality indicators for training programmes; independent sampling and assessment of portfolio work; trainee surveys; and informal feedback from Heads of Schools and advisory committees (Table 9b and c). Interviewees focused on the impact of PCC in the curriculum for trainers and trainees rather than for individual patients or groups of patients (Table 9d). The RCPsych interviewee indicated that most trainers were convinced of the need for training and assessment, whereas trainees struggled more to see the value (Table 9e). Measuring patient experience directly was described to be challenging; it was noted by RCGP, for example, that this was more likely to be measured indirectly through assessment of trainee competence (Table 9f).

**Table 9:** Approaches and attitudes to PCC identified by quality assurance measures

a) “we haven’t got a document that maps the actual competencies to the teaching, training curriculum, we don’t go and quality assure that they’re actually doing it or not” (RCPsych)
b) “the way in which things are done, executed, assessed, etc. is basically agreed by all the TPDs [Training Programme Director], who will be the people who will be implementing it all. And so you get feed-down, and feedback, in that way. < … > So, we basically appraise all our TPDs. There’s an annual report from each TPD about their STC [Specialty Training Committee], and there’s an overarching, as you know, annual report from the School of Medicine. And that’s fed up to PTB [Programme Training Board], and then, eventually, to the GMC.” (RCP)
c) “through the ARCP we also find out whether there is evidence that it is working and where the problem areas are, then of course we still have the JCST [Joint Committee on Specialist Training] training survey which we have done, another way of checking up that things are happening. < … > but we check that if there are any problem areas and what has been done about it” (RCS)
d) “I don’t think we measure patient experience directly, we have looked at training experience and trainer experience” (RCPsych)
e) “I think there was more of a resistance from trainees, < … > most trainers felt that including patient-centred elements in the curriculum and assessment was a good idea, and that we needed to do that, and we needed to improve the way we did it in our training < … > our trainees were probably not so vociferous in their expression of this, but I think some of them clearly felt that < … > this was another tick box exercise” (RCPsych)
f) “the issue of being able to evidence impact came up. This was in respect of the question about the impact of their curriculum on patient experience. He noted that this is quite difficult, and that they are asked to show this in their business case. He did however state that the impact of person-centred care in the programme was seen, he believed, in the training results; i.e. their ability to demonstrate the competencies which they have in the programme. These are based on patient-centred care, communication, and effective use of interpersonal skills” (Fieldnote, RCGP)

## Discussion

This research is the first to consider how PCC is represented in postgraduate medical curricula in the UK, and to explore factors influencing inclusion of PCC in curricula content, using four Royal Colleges as examples (RCGP, RCPsych, RCP, RCS). PCC and associated concepts such as SDM are increasingly described as expected practice among UK healthcare professionals throughout a range of policy documents [1–4, 23, 28, 53, 54] and medical registration requisites [50]. Combined analysis of interviews and curricula documents revealed inconsistent definitions of PCC that lacked specificity, alongside variations in terminology used to identify components of PCC. Noticeably the documents used “patient-centred” rather than “person-centred” care, which in part reflects the variation in definitions identified, but may represent a more limited perspective. as discussed in a comparative analysis of themes within literature using the two terms [55].

PCC was included in the curriculum in different ways; for example, some Colleges teach PCC through professional skills training and some through specific conditions. However, no College included in this study directly monitored how PCC teaching is implemented, allowing significant scope for variation across local deaneries. Quality assurance measures were focused on trainers and trainees, rather than patients, and interestingly, interviewees felt that trainers appeared more bought into the need for PCC teaching than trainees. Moreover, ways that patients and the public can and do contribute to curriculum development, teaching, and assessment of competence lacked clarity and so the meaningfulness of those contributions is uncertain.

Working definitions of PCC and what constitutes a PCC-competent practitioner lacked specificity and consistency across colleges (with RCGP providing the most comprehensive definition), which corresponds to the lack of clarity seen within the literature [56, 57] and professional standards [50]. Recent research indicates that lack of understanding flows through to trainees in different specialities [58]. Since completion of our analysis, the GMC has released the Generic Professional Capabilities Framework [59], which, whilst not including a specific section on PCC, does provide a broad definition in the glossary. While this definition covers the concept of PCC, it does not specify the skills necessary to ensure accurate and consistent application to postgraduate medical training. Attempts have been made to create a competency framework for SDM in the UK [60], and SDM activities and behaviours were recently identified by a range of experts in the Netherlands [61]. Neither identifies competence expectations based on stage of training though, and no such competency framework exists for PCC more broadly. Cross-organisation collaboration to develop a PCC competence framework that defines what skills are expected, as well as the level of competence required at different points in training, would address some of these issues. The Academy of Medical Royal Colleges may have a role to play in coordinating efforts to deliver a consistent approach to PCC training in postgraduate medical education.

A consequence of the current lack of clarity around PCC identified by interviewees was that different groups may have a different understanding of the same terminology. Thus, healthcare professionals may be practicing PCC without directly interpreting it as such, and others outside of the profession may not recognise PCC within the curricula content. While use of the skills is more important than the terminology, this variation can create challenges for auditing PCC content across curricula, evaluating teaching, and for sharing practice between specialties. Indeed, this was evident in the variability across Colleges in how PCC was included in their curriculum, a finding that has also been observed in undergraduate medical education [26]. Some Royal Colleges focused their PCC content on professional skills training, whereas others inserted it within training about specific conditions. This difference in PCC-curricula location may lead to very different types of PCC practitioners, with some differences in flexibility of usage across consultations. Whilst we can observe this difference, we have very little knowledge about which approach leads to more PCC-competent practitioners, and how context may influence best practice in PCC training. Only systematic evaluation and comparison of different teaching methods in different contexts will clarify the point.

A number of national policies, legislations, and guidelines influenced inclusion of PCC in the curriculum (e.g. [50, 51, 62], but a key driver for all colleges was the GMC, which can be seen in some of the commonalities of approach across colleges. One such commonality was a strong emphasis on communication skills, which was also found in undergraduate medical education [26]. The driving force behind this may be the GMC’s Good Medical Practice guidelines [50] which emphasise communication skills and feature heavily in all of the curricular documents analysed, regardless of speciality. Another commonality was PCC in reference to long term conditions and end of life care, even when the general focus was on professional skills. Both the GMC [50], and NHS England [54, 63–65] have developed a number of policy guidelines that support inclusion of PCC in these areas of care, which may again feed into the specifics of PCC curriculum development across the Royal Colleges.

It is important to recognise that while Royal Colleges set curricula content, they do not have direct oversight over the methods or extent to which content is applied by local deaneries, nor the success of approaches. A recent study with postgraduate medical trainees suggested that they perceived very little formal PCC teaching, following conceptual learning from undergraduate education, GMC and Trust guidelines, and that most learning happened through observations on the job [58]. It is unclear whether such perceptions might relate to a lack of recognition about PCC training opportunities, or lack of direct training being utilised. Royal Colleges do use feedback mechanisms for quality assurance, and interviewees described lack of direct summative assessment, differences in assessment methods, along with limited patient experience assessment, and reflections about the success of assessment techniques to holistically evaluate person-centredness. Conversely, RCP had gone to some lengths to improve their PCC assessment, and felt that the results were positive. These factors make assessment of PCC competence amongst trainees, and the success of the curricula in helping trainees to achieve that competence, challenging. Without adequate quality assurance, PCC skills are unlikely to be held to the same rigour as other areas of the curriculum [66]. While greater involvement of Royal Colleges could ensure greater consistency in approaches across local deaneries, there is value in flexibility of local provision to meet local needs, and it is important to strike a balance between the two.

Whilst trainers were clear that PCC training and assessment were important, and could be improved upon, interviewees indicated that some trainees believe it is a tick box exercise, that they already know how to do it, and that it is patronising to suggest that they need training. These attitudes may stem from the fact that PCC encompasses a number of soft skills that are taught throughout undergraduate education, or perhaps from PCC content at postgraduate level. Beliefs about the pre-existing PCC competence of trainees might impact negatively on their willingness to engage meaningfully with existing training [67]. A round-table debate about improving PCC provision suggested that Royal Colleges should lead change in this area by getting trainees to a point where they realise they do not know everything [68], which would hopefully improve engagement with training. The Royal Colleges may also have a role in making PCC training (formal or informal) more readily identifiable by trainees, along with explicitly stating how competence expectations go beyond undergraduate training; a place where a clear competence framework would again play a role. Furthermore, involving trainers in the development of meaningful measures of PCC could provide clinically relevant, practical, and deliverable solutions for embedding PCC training and assessment.

In addition to the value of trainer-input, there is a clear need for greater involvement of patients and the public in PCC curriculum development, teaching and assessment. The limited involvement of patients and the public is particularly concerning given the topic being addressed. Whilst all interviewees reported patient and public representatives on relevant committees, there was only limited evidence of them as active influencers of change. A meta-narrative review [69] around patient involvement in healthcare professional’s education focused on learning using the patient as teacher, which moves beyond biomedical models, the role of real patients as ‘standardised patients’, and learning as part of service provision using constructivist theories of learning. Evidence is sparse regarding the role of patients in curriculum development [69, 70], highlighting a clear need to develop models of inclusion and evaluate best practice in this area. Recently published NICE guidance [71] about organisation-level implementation of SDM, including utilisation of service users, may provide insight into flexible curricula implementation from Royal College through to local deanery.

### Strengths and limitations

These results are only applicable to the Royal Colleges involved in this work, and the curricula documents analysed. Specialist postgraduate medical training routes are (necessarily) complex and so this work focused on core training, with an assumption that this would include PCC. However, during our analyses, we discovered that the RCS specialisations contained a specific section (standardised across all specialities) which covered professional skills and contained more about PCC than their core curriculum. Furthermore, all curricula referenced the GMC’s ‘Good Medical Practice’ [50], suggesting that curricula documents require a broader set of readings and understandings. As such, we relied upon the Royal College interviewees to indicate where we would find reference to PCC, but some PCC content may have been missed by this analysis. Future research exploring the full complexity of one specialisation may be beneficial. In this TNA, we did not consult trainers, trainees or patients, although closing the loop in this manner by exploring how curricula are interpreted in practice by different stakeholders would help inform all aspects of the postgraduate curriculum. It would also be beneficial to extend this work into continuing professional development training, post specialisation.

The strengths of the study include that this is the first such study to explore PCC as driven by UK College curricula and included triangulation between interviews with key informants and a documentary analysis. The analytical framework was informed by a focused literature review and further developed collaboratively by the authors. A range of Colleges were included to reflect different types of speciality (surgical/non-surgical, primary/secondary/mental health care, and colleges with subspecialties). On the other hand, the interviews were conducted with one key informant from each college (albeit selected carefully for their expertise and understanding of the curricula), but this was also countered by the triangulation with College documentation. Future work would benefit from surveying a wider sample across all levels (trainers, trainees, patient and public representatives within the Colleges) to explore whether wider members of the Colleges share these views.

## Conclusion

To conclude, we identified a number of challenges to achieving high quality PCC training at postgraduate level, including lack of clarity about the specific meaning of PCC and the skills required to become a PCC-competent practitioner, variation in implementation and uncertainty about the best ways to teach and assess PCC skills, lack of trainee buy in, and uncertainty about how patients and the public can contribute to curriculum development, teaching, and assessment of competence. Nevertheless, there are legal and policy imperatives demanding healthcare professional competence in PCC, as well as desire by Royal Colleges to understand how to do this more effectively. There is a need for cross-organisation collaboration to develop a PCC competence framework that defines the skills and level required at different points in training. It is important to ensure clarity around the differences between undergraduate and postgraduate PCC requirements, to increase trainee engagement with this aspect of the curriculum. Greater auditing and quality assurance of programme delivery would be beneficial for identifying successful practices to share within and across Royal Colleges, while still maintaining the flexibility of provision provided locally. Engagement with patients and the public in this work can only strengthen provision.

### Supplementary Information

**Additional file 1. **Appendix.

**Additional file 2.** Supplementary Tables.

## Data Availability

The datasets generated and/or analysed during the current study are not publicly available. Data are however available from the corresponding author upon reasonable request and with permission of project participants.
