# Supplementary material for: Inclusion of person-centred care in UK postgraduate medical education curricula: Interviews and documentary analysis
Source: BMC Med Educ. 2023 Oct 11;23:757. doi: 10.1186/s12909-023-04730-2 (PMC10568751; doi:10.1186/s12909-023-04730-2)
Supplement: Supplementary file 1 — Additional file 1. Appendix. [file 12909_2023_4730_MOESM1_ESM.docx]

Postgraduate Training Needs Analysis Interview Questions

**Background information**

The Health Foundation and Health Education North East (HENE) are jointly funding the third phase of the MAGIC programme (Making Good decisions In Collaboration – <http://www.health.org.uk/areas-of-work/programmes/shared-decision-making/>), which aims to support implementation of shared decision making (SDM) and person-centred care in selected Trusts across the North East.

As part of this work, we are exploring how national policy on shared decision making and person-centred care is interpreted and integrated into educational curricula. At present this involves working with Royal Colleges to map current content and delivery of postgraduate education and training with respect to shared decision making and person-centred care (addressing what has been called “skills for 21st century practice”).

Ultimately, this training needs analysis will help to inform future commissioning and delivery of education and training for shared decision making and for 21st century person-centred care.

I’d like to ask you some questions first about yourself and your job role, then about your education programme, followed by some questions about how person centred care and shared decision making are implemented in your field.

**Role of interviewee**

1. What is your responsibility in relation to curricula development?

**Demographics**

I’d like to ask you a few questions about your programme to get some background information.

1. When was your curricula last reviewed?
2. How many trainees are undertaking speciality training with your College?
3. What impact is current national/regional policy having on having on your curricula in relation to person-centred care?
4. As a College, are you considering or are you currently driving/leading/initiating changes to curricula in respect of person-centred care?

**Person-Centred Care**

Now I’d like to move on and ask you a few questions about how person-centred care is included in your programme. In this work we are very broadly defining person-centred care as care which is compassionate, personalised, coordinated and enables people to develop their strengths in order to live independent and fulfilling lives. It is a holistic approach that considers not only the condition but also the patient’s preferences, social situation, background and lifestyle. Within person-centred care are multiple facets including shared decision making (SDM), supported self-management (SSM), personalised care planning (PCP), management of long term care (LTC), and communication skills.

1. Considering person-centred care as a whole, and also specific facets (e.g. SDM, SSM, PCP, LTC, communication skills); could you describe which of these your curricula includes and how they are covered?
2. Do you provide any guidance or stipulate on how person-centred care should be taught and/or assessed?
3. How do you incorporate these standards into your professional exams?
4. Have there been any challenges to inclusion of person-centred care in your curricula and exams?
5. What impact has inclusion of person-centred care in your curricula had in terms of practice or patient experience?
6. Is there anything else that you would like to comment on with respect to person-centred care in postgraduate education?

**Request for Curricula Content**

It would be useful for us to see examples of person-centred care in your curricula. If you have any further information other than the published curricula documents on the Royal College website, it would be really helpful to see this as it would allow us to undertake a more detailed analysis.

I am looking for documentation, such as:

- Study guides
- Learning materials
- Assessment materials
- Assessment criteria
- Course evaluation materials
- Policy guidance
- Published reports
