# Supplementary material for: Inclusion of person-centred care in UK postgraduate medical education curricula: Interviews and documentary analysis
Source: BMC Med Educ. 2023 Oct 11;23:757. doi: 10.1186/s12909-023-04730-2 (PMC10568751; doi:10.1186/s12909-023-04730-2)
Supplement: Supplementary file 2 — Additional file 2. Supplementary Tables. [file 12909_2023_4730_MOESM2_ESM.docx]

Supplementary Table 1. Framework used for interview analysis.

| Framework Code | Description |
| --- | --- |
| Inclusion of PCC | How and when PCC is incorporated into the curriculum (including components of PCC, e.g. shared decision making, personalised care planning, etc.). |
| Influences on the curriculum | The factors that influence curriculum content generally, and in relation to PCC specifically. |
| Barriers and Facilitators to Inclusion | Difficulties associated with including PCC in the curriculum or factors that aid inclusion. |
| Quality Assurance | How Colleges know that PCC is taught and the impact that this has on clinicians and service users. |
| Patient and Public Involvement | The role of patients, carers and the public involved in the curriculum (influencing content AND in teaching/assessment of trainees). |
| The nature of PCC* | How PCC is understood by interviewees. |

** This theme was not part of the initial framework coding but emerged from the data.*

Supplementary Table2. Framework used for curricula analysis.

| Theme | Description |
| --- | --- |
| Planning | Personalised care planning; Care and support planning; Collaborative care and support planning |
| Coordination | Coordinated care; Effective and efficient coordination of care; Strengthening multi-disciplinary care teams |
| Shared decisions | Shared decision making; Share decisions about treatment with patients/ families/ carers; Prompting people to be more engaged in health consultations |
| Health literacy | Helping people to learn more about their conditions; Increasing health literacy |
| Self-management | Self-management support; Information, education and support for self-management; Improving capacity for self-management and self-care |
| Communication | Clear and effective communication between health professionals and patients/ carers/ families; Non-discriminatory and culturally appropriate/ sensitive and accessible communication; Treat patients as whole beings not just conditions |
| Involvement | Involving patients/ carers/ families in services and service planning |
